# Value of faecal exfoliated cells in colorectal tumour screening using SDC2 methylation test

**DOI:** 10.1080/07853890.2023.2261111

**Published:** 2023-10-02

**Authors:** Yan-Mei Liu, Lei Peng, Chen Chen, Peng Zhou, Bin Cheng, Ying Luo, Mei-Fang Zhou, Shu-Xia Xuan, Jin-Duan Lin, Wei-Guo Yin

**Affiliations:** Department of Laboratory Medicine, The Sixth Affiliated Hospital of Guangzhou Medical University, Qingyuan People’s Hospital, Qingyuan, People’s Republic of China

**Keywords:** Colorectal tumour, methylation, mSDC2

## Abstract

**Background:**

This study aimed to evaluate the diagnostic value of a non-invasive methylation gene test in clinical colorectal tumour screening.

**Method:**

The quantitative methylation-specific PCR technique was used to detect faecal methylated syndecan-2 (mSDC2) in patients who received the screening of colorectal cancer (CRC).

To evaluate the positive predictive value (PPV) of mSDC2 in patients with colorectal cancer, advanced adenoma (AA), and colorectal tumor (CRN) in risk factor stratification.

**Results:**

The PPV of CRC, CRC + AA and CRN in male patients were 28.03%, 43.55% and 56.24%, respectively, which were higher than female patients. The positive detection rate of mSDC2 and the PPV of CRC gradually increased with age; The PPV in patients aged over 80 years was up to 78.05%, which was more significant than in younger patients with CRC. The PPV of CRC, AA and CRN were 37.10%, 11.80% and 63.37%, respectively. mSDC2 has a high detection rate of 85–100% in AA with intramucosal carcinoma alone or in combination with severe atypical hyperplasia or villous adenoma.

**Conclusion:**

The mSDC2 test has a higher PPV in patients with colorectal cancer and colorectal adenoma (AD), especially in high-risk groups over 50 years of age, and may help in the early diagnosis of colorectal tumours in the future.

## 1. Background

Colorectal cancer (CRC) is a malignant lesion of the colorectal mucosa caused by various environmental or genetic factors, including colon and rectal cancer. It is one of the most common gastrointestinal malignancies worldwide. The number of new cases of bowel cancer in China was 592,232, with 309,114 deaths in 2022, ranking fifth in the tumour category, and the incidence and mortality rates are increasing every year [1]. Most of CRCs are formed from AD progression, and it takes 3–10 years from AD to CRC, providing valuable and sufficient screening time for the early diagnosis of CRC [2]. Traditional detection methods commonly used include faecal occult blood testing, colonoscopy and computer tomography, but all the aforementioned technologies have shortcomings, such as low specificity or sensitivity, high invasiveness and high radiation, which make CRC screening difficult. Thus, it is important to explore tests with high sensitivity, non-invasive and better compliance to achieve early screening of CRC [3–6].

Syndecan-2 (SDC2) is a fibroglycan belonging to the transmembrane (type I) acetyl heparan sulphate proteoglycan family. Studies have shown that SDC2 is hypermethylated in malignant glioma tumour tissues [7]. Methylated syndecan-2 (mSDC2) has also been detected in the blood of patients with CRC [8]. Since the exfoliation of tumour cells into the colorectal lumen precedes vascular infiltration during the development of CRC [9], faeces are theoretically more suitable than blood tests for the early detection of colorectal tumours. Non-invasive faecal genetic screening for patients with CRC is currently being conducted at our hospital. Quantitative methylation-specific PCR was used to detect mSDC2 in faecal specimens to assess the risk of colorectal tumour development. Specimen collection is simple, convenient and can be completed at home without invasive risks. In this study, the clinical value of this test as an early screening method for colorectal tumours was further evaluated by measuring mSDC2 levels in faecal specimens of patients using colonoscopy, computer tomography imaging and pathological tests.

## 2. Study protocol

### 2.1. Scope of the study

Patients who visited Qingyuan People’s Hospital for faecal exfoliated cell mSDC2 testing from March 2017 to February 2022 had their faecal specimens collected under the guidance of laboratory professionals and agreed to subsequent follow-up. This study was approved by the Ethics Committee of the Sixth Hospital of Guangzhou Medical University (Qingyuan People’s Hospital; Approval No. IRB-2021-023).

### 2.2. Study population

All records for each patient tested for faecal mSDC2 were extracted from the Laboratory Information (LIS), outpatient, physical examination and inpatient systems. An auditor reviewed the records to ensure data accuracy.

The overall screening population was divided into an elderly group aged ≥50 years and a younger group aged <50 years, according to the 2020 Chinese Society of Clinical Oncology Diagnosis and Treatment Guidelines for Colorectal Cancer and the Expert Consensus on Early Colorectal Cancer Screening Process in China published in Shanghai in 2019 [10]. The target population was then divided into physical examination and high-risk populations by risk stratification according to the clinical diagnosis of the LIS system.

Target population: (1) Health check-ups; (2) high-risk factors for bowel cancer (with any of the following high-risk factors): (a) symptoms related to chronic constipation, chronic diarrhoea, bloody stools, or changes in stool habits; (b) elevated CEA or CA19-9; (c) chronic gastric diseases, gastrointestinal neurosis or gastric tumours; (d) chronic intestinal diseases or tumours to be investigated; (e) anal diseases or tumours; (f) history of chronic appendicitis or appendectomy; (g) history of chronic biliary tract disease or cholecystectomy; (h) tumour at other sites, such as breast cancer, pancreatic cancer, etc.

Exclusion criteria: Patients (1) whose sampling time was the same day as the colonoscopy, (2) with a CT value of the internal reference gene ACTB of mSDC2 gene test over 36, (3) were tested repeatedly, (4) were tested after bowel cancer surgery, (5) with a history of CRC and (6) with unrelated diseases such as type 2 diabetes, lumbar disc herniation, chronic lymphadenitis, thrombocytopenia, liver disease, abdominal hernia and oesophageal disease.

### 2.3. Clinical treatment

#### 2.3.1. Faecal mSDC2 test

Around 4.5 g of formed faeces was placed in a sampling tube containing 16 ml of protective solution. DNA fragments of the target gene SDC2 and the internal reference gene ACTB were extracted from the faeces using the magnetic bead method and then treated with 25% sulphite to convert the unmethylated cytosine into uracil (‘Chang’an Xin’ Bowel Cancer Faecal Gene Test Kit, Kang Li Ming Company, China). Ten microliters of the purified product were transferred for PCR. Conditions included denaturation at 95 °C for 5 min; 48 cycles (95 °C for 15 s, 58 °C for 30 s, 72 °C for 30 s) and then cooling at 40 °C for 30 s. *In vitro* mDNA was used as a positive control and normal human peripheral blood lymphocyte DNA was used as a negative control. Results were determined based on the CT value: (1) CT value of ACTB gene ≤36 indicates that the specimen is qualified, CT > 36 means that the specimen is not qualified and needs to be resampled or rejected; (2) CT value of mSDC2 gene ≤38 means positive, indicating hypermethylation of SDC2; and CT value >38 or no product amplification is negative, indicating hypometh­ylation or no methylation of SDC2. For statistical purposes, the CT of those without the mSDC2 amplification products was set to 48.

#### 2.3.2. Subsequent follow-up examinations and pathological diagnosis

Patients further underwent colonoscopy, and those who could not undergo colonoscopy due to age and disease underwent CT-enhanced imaging of the abdomen to record lesion size, shape and location (lesions located within 60 cm from the anus were considered distal, mainly including the rectum, sigmoid colon and partial descending colon; lesions located >60 cm from the anus were considered proximal) [11]. All patients with a definite diagnosis were examined by colonoscopy or postoperative pathology for adenocarcinoma (CRC), advanced adenoma (AA; ADs ≥1 cm in diameter or >25% villous or severely atypical hyperplastic AD or serrated AD and diameter ≥1 cm), colorectal neoplasm (CRN; including AD, AA and adenocarcinoma) [12], polyps and normal (non-neoplastic, non-polyposis intestinal disease, as indicated by the colonoscopy report showing no abnormality, enteritis and diverticulosis colon). According to the 2021 NCCN Clinical Practice Guidelines for Colorectal Cancer Oncology [13], patients with CRC with complete electronic colorectal and pathological examinations were classified into stages.

### 2.4. Statistical methods

Data were input using Microsoft Excel 2016, and statistical analysis and plotting were performed using GraphPad Prism 8 software. Differences in rates and correlations between the groups were compared using the chi-square test of cross-tabulation with rows multiplied by columns or Fisher’s exact test. Pearson’s correlation coefficient was used to analyse the correlation between the positive predictive value (PPV) of the SDC2 methylation test for colorectal tumours and age. The Kruskal–Wallis rank sum test was used to compare the overall differences in CT values between multiple groups. The difference was considered statistically significant when *P* < 0.05, and two-sided test level *α* = 0.05.

## 3. Results

### 3.1. Total population

**Figure 1.**
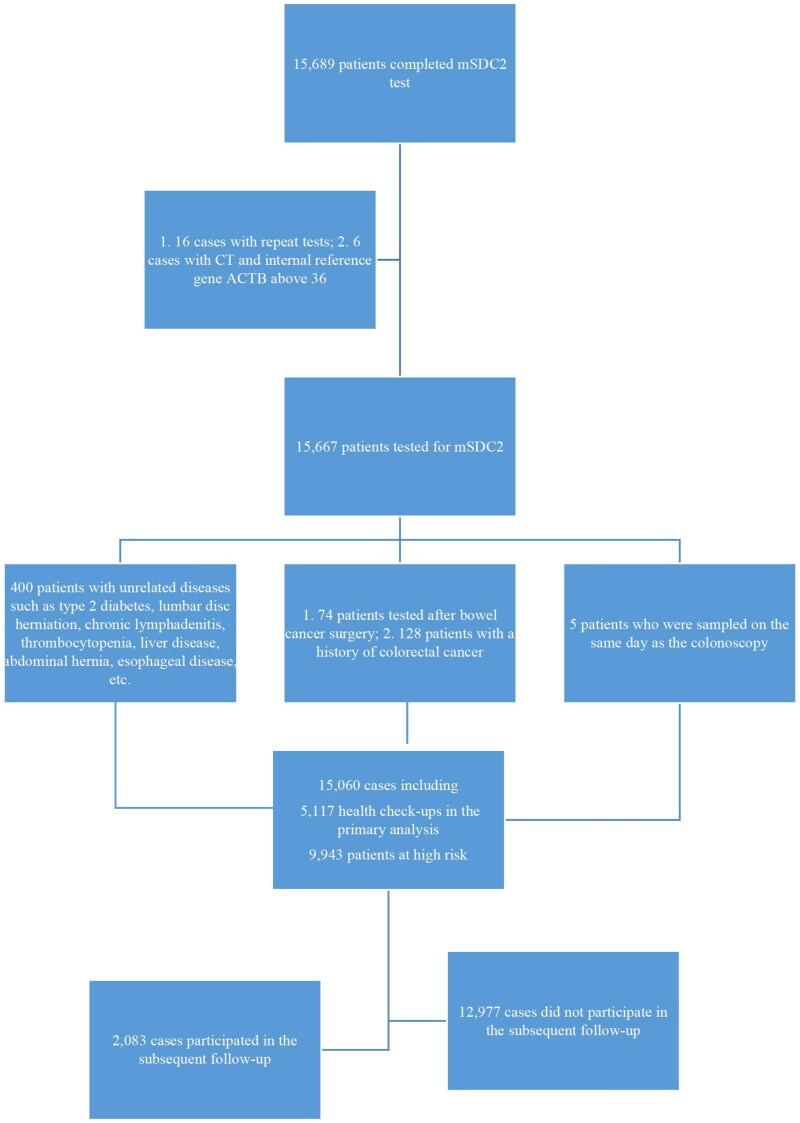
Total population size and results.

A total of 15,060 patients were finally included in this study for analysis, including 9,943 patients in the high-risk group for CRC and 5,117 patients in the physical examination group (Figure 1). The mSDC2 positive cases in the high-risk group were 932 (9.37%), significantly higher than that in the physical examination group (4.06%). The positive detection rate of mSDC2 in the high-risk group was significantly higher than that in the physical examination group (10.66% vs. 4.43% in males and 8.12% vs. 3.47% in women, respectively; Table 1). The mSDC2 positive detection rate increased gradually with age in the high-risk and physical examination groups, and the mSDC2 positive detection rate was higher in the high-risk group than in the physical examination group at all ages. The differences between the remaining groups were statistically significant, except for the groups aged <20 and ≥80 years (Table 1).

**Table 1.** Detection of mSDC2 test in the high-risk and physical examination groups based on gender and age.

	Total	High-risk group			Physical examination group			*χ*²	*P*
		Total	Positive (*n*%)	Negative (*n*%)	Total	Positive (*n*%)	Negative (*n*%)		
Total	1,560	9,943	932 (9.37)	9,011 (90.63)	5,117	208 (4.06)	4,909 (95.97)	136.071	<0.0001
Gender									
Male	8,077	4,917	524 (10.66)	4393 (89.34)	3,160	140 (4.43)	3,020 (95.57)	98.848	<0.0001
Female	6,983	5,026	408 (8.12)	4618 (91.88)	1,957	68 (3.47)	1,889 (96.53)	47.806	<0.0001
Age									
<20	122	102	2 (1.96)	100 (98.04)	22	0 (0)	22 (100)	–	–
20–29	1,195	984	28 (2.85)	956 (97.15)	240	1 (0.42)	239 (99.58)	4.921	0.0265
30–39	3,018	2,171	103 (4.74)	2068 (95.26)	960	10 (1.04)	950 (98.96)	26.233	<0.0001
40–49	3,548	2,315	160 (6.91)	2155 (93.09)	1,420	27 (1.90)	1,393 (98.10)	46.450	<0.0001
50–59	3,560	2,351	238 (10.12)	2113 (89.88)	1,519	72 (4.74)	1,447 (95.26)	36.293	<0.0001
60–69	1,668	1,274	197 (15.46)	1077 (84.54)	650	59 (9.08)	591 (90.92)	15.217	<0.0001
70–79	823	577	148 (25.65)	429 (74.35)	246	27 (10.98)	219 (89.02)	22.183	<0.0001
≥80	229	169	56 (33.14)	113 (66.86)	60	12 (20.00)	48 (80.00)	3.660	0.0557

### 3.2. Follow-up after faecal mSDC2 gene test

Of the 15,060, 12,977 patients did not undergo follow-up examinations, and only 2,083 patients underwent colonoscopy, rectal MRI, or abdominal CT-enhanced imaging, which included 1,806 patients in the high-risk group and 277 patients in the physical examination group. A total of 682 patients in the high-risk group and 116 patients in the physical examination group tested positive for mSDC2, with follow-up examination perfection rates of 73.18% (682/932) and 55.77% (116/208), respectively. In addition, 1,124 patients in the high-risk group and 161 patients in the physical examination group with negative mSDC2 results participated in the follow-up examination, with perfection rates of 12.47% (1124/9011) and 3.28% (161/4909), respectively.

### 3.3. mSDC2 test in different risk population

Patients were divided into groups aged ≥50 years and <50 years and into high-risk and physical examination groups based on the clinical diagnosis (Table 2). Among the 1,106 patients in the group aged ≥50 years, 176 patients with CRC, 56 with AA, 232 with CRC + AA and 301 with CRN were detected in the high-risk group of patients with positive mSDC2, with detection rates of 37.10%, 11.80%, 48.90% and 63.37%, respectively. Among the negative patients, 32 cases of CRC, 38 cases of AA and 168 cases of CRN were diagnosed, with omission rates of 5.10%, 6.00% and 37.72%, respectively. Fourteen patients with CRC, 18 with AA, 32 with CRC + AA and 51 with CRN were detected in the physical examination group of patients with mSDC2 positivity, with detection rates of 14.70%, 18.90%, 33.60% and 53.68%, respectively. Three cases of CRC, 5 cases of AA and 23 cases of CRN were diagnosed among the negative patients, with omission rates of 3.40%, 5.70% and 26.44%, respectively. The detection rates of CRC in the high-risk group of patients with positive mSDC2 were significantly higher than those in the physical examination group, and the differences were statistically significant (*χ*^2^ = 97.449, *P* < 0.001). The detection rates of AA and AD in the healthy group were significantly higher than those in the high-risk group (*χ*^2^ = 321.238, *P* < 0.001; *χ*^2^ = 162.797, *P* < 0.001).

**Table 2.** Pathology results in the high-risk and physical examination groups aged ≥50 years.

Total	High-risk group			Physical examination group			*χ*²	*P*
	Total	Positive (*n*%)	Negative (*n*%)	Total	Positive (*n*%)	Negative (*n*%)		
Total	1,106	475	631	182	95	87	–	–
CRN	539	301 (63.37)	238 (37.72)	74	51 (53.68)	23 (26.44)	–	–
CRC	208	176 (37.10)	32 (5.10)	17	14 (14.70)	3 (3.40)	97.449	<0.001
AA	94	56 (11.80)	38 (6.00)	23	18 (18.90)	5 (5.70)	321.238	<0.001
AD	237	69 (14.50)	168 (26.60)	34	19 (20.00)	15 (17.20)	162.797	<0.001
Negative for all non-AA, non-neoplastic manifestations and colonoscopy	567	174 (36.70)	394 (62.20)	108	44 (46.30)	64 (73.50)	–	–
Polyps	122	34 (7.20)	88 (13.90)	27	14 (14.70)	13 (14.90)	332.592	<0.001
Normal	445	140 (29.50)	305 (48.30)	81	30 (31.60)	51 (58.60)	178.531	<0.001

*Note:* CRC: colorectal cancer; CRN: colorectal neoplasia; AA: advanced adenoma; AD: adenoma.

Among the 700 patients in the group aged <50 years (Table 3), 29 cases of CRC, 15 cases of AA, 44 cases of CRC + AA and 61 cases of CRN were detected in the high-risk group of patients with positive mSDC2, with detection rates of 14.01%, 7.25%, 21.26% and 29.47%, respectively. Seven cases of CRC, 17 cases of AA and 89 cases of CRN were diagnosed in negative patients, with omission rates of 1.42%, 3.45% and 18.05%, respectively. One case of CRC, three cases of AA, four cases of CRC + AA and five cases of CRN were detected in the physical examination group of patients with positive mSDC2, with detection rates of 4.76%, 14.29%, 16.67% and 23.81%, respectively. One case each of CRC and AA and 13 cases of CRN were diagnosed in the negative patients, with omission rates of 1.35%, 1.35% and 17.57%, respectively. The CRC detection rate in the high-risk group of patients with positive mSDC2 was significantly higher than in the physical examination group, and the difference was statistically significant (*χ*^2^ = 86.963, *P* < 0.001; Table 3).

**Table 3.** Pathology results in the high-risk and the physical examination groups aged <50 years.

Total	High-risk group			Physical examination group			*χ*²	*P*
	Total	Positive (*n*%)	Negative (*n*%)	Total	Positive (*n*%)	Negative (*n*%)		
Total	700	207	493	95	21	74	–	–
CRN	150	61 (29.47)	89 (18.05)	18	5 (23.81)	13 (17.57)	–	–
CRC	36	29 (14.01)	7 (1.42)	2	1 (4.76)	1 (1.35)	86.963	<0.001
AA	32	15 (7.25)	17 (3.45)	4	3 (14.29)	1 (1.35)	309.746	<0.001
AD	82	17 (8.21)	65 (13.18)	12	1 (4.76)	11 (14.86)	178.413	<0.001
Negative for all non-AA, non-neoplastic manifestations and colonoscopy	550	146 (70.53)	404 (81.95)	77	16 (76.15)	61 (82.43)	–	–
Polyps	88	29 (14.01)	59 (11.97)	12	4 (19.05)	8 (10.81)	173.990	<0.001
Normal	462	117 (56.52)	345 (69.98)	65	12 (57.10)	53 (71.62)	168.819	<0.001

*Note:* CRC: colorectal cancer; CRN: colorectal neoplasia; AA: advanced adenoma; AD: adenoma.

### 3.4. mSDC2 test in AA

Among 153 patients with AA (Table 4), five cases of serrated AD were detected and two cases were missed, with a detection rate of 60%. 58 cases with ADs ≥1 cm in diameter were detected, of which 27 cases had a detection rate of 46.55%, while 31 cases were missed. 62 cases of villous tubular AD and/or severe atypical hyperplasia and/or intramucosal carcinoma were detected, with a detection rate of 68.89%, while 28 cases were missed. The detection rate of simple intramucosal carcinoma and/or severe atypical hyperplasia and villous tubular AD was high, followed by severe atypical hyperplasia and AA (78.57%). Therefore, the detection rate of mSDC2 was significantly higher in simple intramucosal carcinoma or compound AA, and the detection was not obvious in mild and moderate atypical hyperplasia ADs with diameters ≥1.

**Table 4.** Performance of SDC2 gene test in different types of AA.

	Total	Positive	Negative
Total	153	92 (60.13)	61 (39.87)
≥1 AD	58	27 (46.55)	31 (53.45)
SSP	5	3 (60)	2 (40)
Others	90	62 (68.89)	28 (31.11)
Villous tubular AD	23	12 (52.17)	11 (47.83)
Villous tubular AD combined with severe atypical hyperplasia	18	11 (61.11)	7 (38.89)
Villous tubular AD combined with severe atypical hyperplasia and intramucosal carcinoma	20	17 (85)	3 (15)
Severe atypical hyperplasia/intramucosal carcinoma	8	4 (50)	4 (50)
Severe atypical hyperplasia	14	11 (78.57)	3 (21.43)
Intramucosal carcinoma	6	6 (100)	0 (0.00)
Villous tubular AD/intramucosal carcinoma	1	1 (100)	0 (0.00)

*Note:* SSP: sessile serrated polyp.

### 3.5. Gender and age distribution for PPV of mSDC2 in colorectal tumours

The PPV of the SDC2 methylation test for males with CRC was 28.03%, which was higher than that for females (23.46%). The PPV of mSDC2 was higher in males than in females in all groups with colorectal tumours. The PPV of males in CRC + AA was 43.55% and in CRC + AA + AD it was 56.24%, which was higher than that of females (33.14% and 42.28%, respectively), with statistically significant differences (*χ*^2^ = 6.946, *P* = 0.008 < 0.05; *χ*^2^ = 12.36, *P* < 0.001; Table 5).

**Table 5.** Differences in PPV distribution of mSDC2 on colorectal tumours based on gender and age.

Parameters	Total number of positive SDC2	CRC	AA	AD	CRC + AA	CRN	*χ*²	*P*
		*n* (PPV%)	*n* (PPV%)	*n* (PPV%)	*n* (PPV%)	*n* (PPV%)		
Total PPV	798	220 (28.03)	92 (11.72)	106 (13.50)	312 (39.75)	418 (53.25)		
Gender								
Male	457	140 (30.63)	59 (12.9)	68 (14.88)	199 (43.54)	257 (56.24)	197.485	<0.001
Female	341	80 (23.46)	33 (9.68)	38 (11.14)	113 (33.14)	151 (44.28)	123.299	<0.001
Age range								
<20	1	0	0	0	0	0	–	–
20–29	12	1 (8.30)	1 (8.30)	0	2 (16.67)	2 (16.67)	2.431	0.85
30–39	81	8 (9.90)	3 (3.70)	7 (8.60)	11 (13.58)	18 (22.22)	15.213	0.002
40–49	134	21 (15.67)	14 (10.45)	11 (8.21)	35 (26.12)	46 (34.33)	52.183	<0.001
50–59	238	41 (17.23)	43 (18.07)	37 (15.55)	84 (35.29)	121 (50.84)	78.758	<0.001
60–69	176	58 (32.95)	21 (11.93)	35 (19.89)	79 (44.89)	114 (64.77)	54.15	<0.001
70–79	115	59 (51.30)	9 (7.83)	15 (13.04)	68 (59.13)	83 (72.17)	44.856	<0.001
≥80	41	32 (78.05)	1 (2.44)	1 (2.44)	33 (80.49)	34 (82.93)	14.392	<0.001

The PPV of mSDC2 in patients with CRC increased with age, with a PPV of 78.05% in ≥80 patients. The PPV of the SDC2 methylation test was more significant in middle-aged and elderly CRC patients. The PPV of AA and AD was not positively correlated with age and were highest in the group aged 50–69 years, whereas the PPV of mSDC2 in CRC + AA and CRN increased with age (Table 5). Therefore, PPV showed an increasing trend with age in all groups except the AA and AD groups, and the PPV of the SDC2 methylation test for colorectal tumours showed an increasing trend.

### 3.6. mSDC2 methylation levels among different pathological types

**Figure 2.**
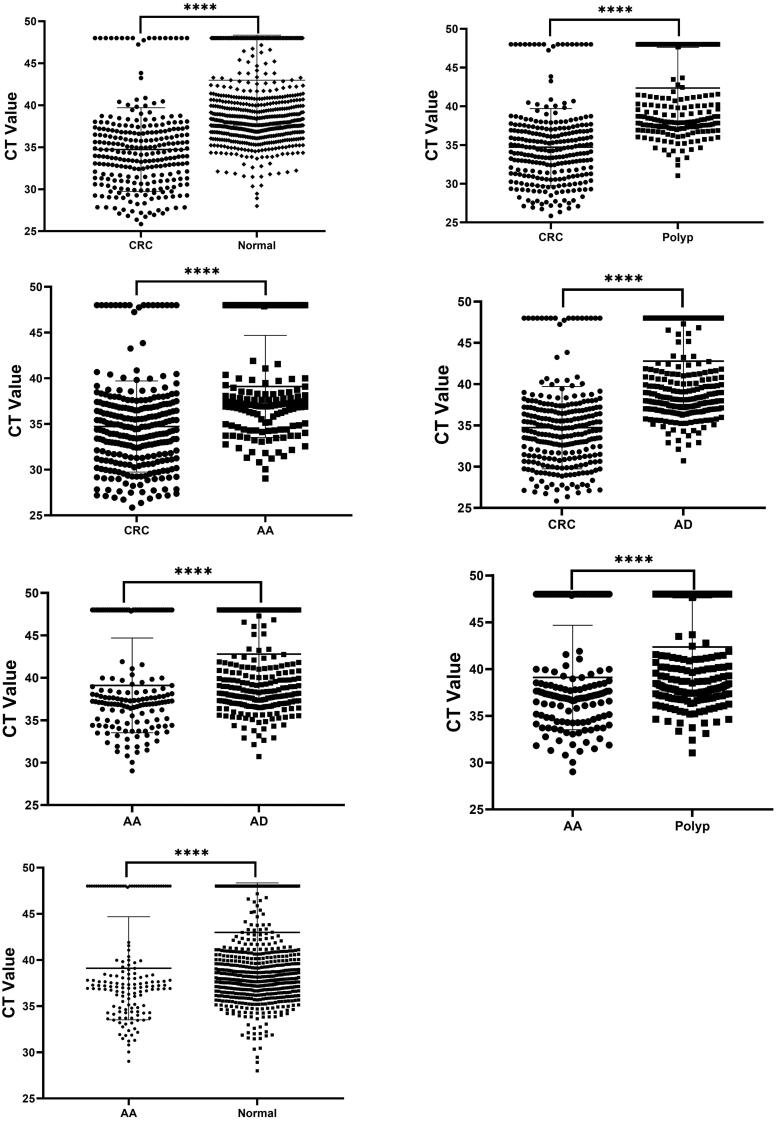
CT values of mSDC2 between groups. *Note:* **** indicates the CT value comparison between groups with *P* < 0.0001.

The cycle threshold (CT) of the mSDC2 test was different between the groups (see Figure 2), with the CT of mSDC2 in normal patients at (46.74 ± 5.36), in polyp-­detected patients at (41.03 ± 5.26), in AD-detected patients at (42.42 ± 5.23), in AA-detected patients at (37.36 ± 5.58) and in CRC-detected patients at (34.35 ± 4.98). As the disease progressed from AD to AA and then to CRC, the CT values of the patients tended to decrease, with the lowest CT values observed in patients with CRC. The difference in the mean CT values between the remaining groups was statistically significant (*P* < 0.001). The CT values of AA were slightly higher than those in the CRC group, and lower than those in the AD, polyp and normal groups. The difference in the CT values between the groups was statistically significant (*H* = 325.9, *P* < 0.001). The difference in CT values was not statistically significant between the AD, polyp and control groups. Lower CT values were more significant in CRC patients.

### 3.7. Intergroup comparison of various clinical characteristics of CRC patients with different degrees of SDC2 methylation

The number of patients diagnosed with CRC after the sequential mSDC2 test and colonoscopy was 263, of which 220 patients were positive for mSDC2 and 43 were negative (Table 6). The detection rate of bowel cancer was 86.96% in 161 male patients and 78.43% in 102 female patients, indicating that the detection rate of bowel cancer was higher in males than in females. A total of five cases were detected in the age groups of 30–95 years and 40–49 years, with a detection rate of 19.20%. The detection rate of bowel cancer in the remaining age groups was above 70%, with the highest rate being 97.0% at ≥80 years. The difference in the detection rate of bowel cancer between the age groups was statistically significant (*χ*^2^ = 12.390, *P* = 0.030 < 0.05), which indicated that in the age group of 40–49 years, the false negative rate for mSDC2 was significant. The positive rate differences among the clinical differentiation, stage and lesion distance groups were not statistically significant. Middle-aged and elderly patients with CRC had a greater positive rate of mSDC2.

**Table 6.** Comparison of clinical characteristics in CRC patients with different degrees of SDC2 methylation.

Parameters	Number of diagnosed cases	SDC2 positive	SDC2 negative	*χ*²	*P*
	(263 cases)	(220 cases) (%)	(43 cases)		
Gender					
Male	161	140 (86.96)	21	3.318	0.069
Female	102	80 (78.43)	22		
Age range					
30–39	10	8 (80)	2	12.390	0.030
40–49	26	5 (19.20)	21		
50–59	51	41 (80.4)	10		
60–69	77	58 (75.3)	19		
70–79	64	59 (92.2)	5		
≥80	33	32 (97.0)	1		
Degree of differentiation					
Low differentiation	33	29 (87.9)	4	1.010	0.962
Moderate differentiation	140	116 (82.9)	24		
High differentiation	8	7 (77.5)	1		
Mucinous adenocarcinoma	9	7 (77.8)	2		
Signet-ring cell carcinoma	1	1	0		
Unknown	72	60 (83.3)	12		
TNM stage					
I	18	14 (77.8)	4	3.762	0.439
II	72	64 (88.9)	8		
III	79	62 (78.5)	17		
IV	42	35 (83.3)	7		
Unknown	52	45 (86.5)	7		
Lesion location					
Proximal end	94	75 (79.8)	19	0.602	0.449
Distal end	147	126 (85.7)	21		
Unknown	22	19 (86.4)	3		

## 4. Discussion

Colonoscopy can reduce the incidence and mortality rates of CRC by 67% and 65%, respectively [14]. The incidence of CRC is decreasing at a rate of 3% per year in the United States due to the high rates of perfect colonoscopy and resection of precancerous lesions [15]. The overall participation rate of colonoscopy in high-risk CRC populations in urban areas in China is insufficient, at only 15.3% [16]. In our study, the overall number of people who underwent faecal mSDC2 test for the first time in 5 years was 15,060 with a positive detection rate of 9.37% in the high-risk group, which was significantly higher than that in the physical examination group (4.06%). The compliance rates of colonoscopy were 73.18% in the high-risk group and 55.77% in the physical examination group, which was significantly improved, indicating that the mSDC2 test helped to improve the compliance of colonoscopy, which can be beneficial in reducing the morbidity and mortality associated with CRC. However, 250 positive patients in the high-risk group and 92 positive patients in the physical examination group did not undergo further colonoscopy, the reasons are listed below: (1) some patients were too old to tolerate the examination; (2) some patients had more underlying diseases and could not tolerate the examination and the risk of anaesthesia and (3) some patients thought they were healthy and did not need the examination.

In our study, the PPV of CRC + AA in male patients using the mSDC2 test reached 43.55% in the total population with high PPV of colorectal malignancies and 33.14% in female patients using CRC + AA, which may be related to the high incidence of colorectal malignancies because males are at higher risk for colorectal malignancies, and also indicates that the mSDC2 test has a high PPV in both male and female patients. The positive detection rate of mSDC2 in males was 10.66% and 4.43% in the high-risk group and physical examination group, respectively, both of which were higher than 8.12% in the high-risk group and 3.47% in the physical examination group in females, which is consistent with the data published in the GLOBOCAN 2020 database [17], where the risk was higher in both males than females, and the crude incidence ratio between males and females was the highest in Asia (1.38:1). It was found that the AA in male patients with the mSDC2 test was higher than that in female patients, suggesting that the SDC2 methylation test has some PPV for AA, and it was higher in males. This may be related to the fact that the male sex is an independent risk factor for the onset of AA [18] and the oestrogen receptor present in female patients can bind to the tumour suppressor genes to inhibit the growth of adenomatous polyps *in vivo* and reduce the incidence of colorectal tumours [19].

Accumulated evidence shows that epigenetic gene regulation is closely related to tumourigenesis, and it has been reported that the positive detection rate of the mSDC2 test in different age groups ranged from 2.2% to 29.7%, and showed a trend for increasing relationship between the methylation rate of the SDC2 gene and the PPV value for CRC, especially in the population above 50 years of age. The PPV increased multifold and was 51.30% in the group aged 70–80 years, while their CRN was 72.17%, indicating that the PPV of the mSDC2 test was even more significant in elderly patients with a high prevalence of CRC [20]. The studies by Sievers et al. [21] showed that the average growth rate of AA was 68% compared to 18% for AD and that AA was more rapid in progression to CRC with fewer detections, which may be the reason for its low PPV in the present study. In 153 patients with AA, the mSDC2 test showed a higher detection rate in serrated ADs (60%) and low detection rates in ADs with mild and moderate atypical hyperplasia of ≥1 cm, but showed a high detection rate of more than 68% in ADs with high atypical hyperplasia and villous percentage of over 25%, especially in ADs with intramucosal carcinoma lesions with a detection rate of 100%. This indicated that the detection rate of the mSDC2 test in AA was related to the pathological changes in AA and a higher detection rate in phase I/II early CRC, indicating that the mSDC2 test can be used for early detection of CRC.

Through further comparison of the PPV differences between the high-risk and physical examination groups aged ≥50 and <50 years, it was found that the mSDC2 test had a significantly higher PPV in CRC and AA in the high-risk group than in the physical examination group aged ≥50 and <50 years. Thus, the mSDC2 test had a more prominent PPV in CRC and AA in the high-risk group but still had some predictive value for CRC and AA in the physical examination group. It should be noted that the mSDC2 test also had PPVs of 21.26% and 19.05% for CRC + AA in the young high-risk and physical examination populations aged <50 years, respectively. Therefore, the lower PPV of the mSDC2 test in the physical examination group aged ≥50 years and younger patients aged <50 years with colorectal tumours may be related to the low incidence in the physical examination and younger age groups. Meanwhile, the study population was divided into a physical examination and a high-risk population based on clinical diagnosis according to the recommended guidelines. The physical examination group was from the medical examination centre of our hospital and the health examinations of those who visited various speciality departments. Patients with high-risk factors were classified based on the physical examination group, which may have caused some bias. The high-risk population was mostly from general surgery, gastroenterology, and other specialized departments. Since the high-risk population was classified by medical history information, patients with relatively low and intermediate risk of colorectal tumours may have been included along with the high-risk population in this study during data screening, which meant that these patients were not strictly at high risk for colorectal tumours.

The positive rate of the mSDC2 test did not differ between the groups in the degree of differentiation, TNM stage and location of lesions in patients with CRC, which is consistent with the results of Wang et al. [22] Feng Niu et al. [23] reported that SDC2 was highly methylated in the faeces of patients with intestinal cancer and less methylated in the faeces of normal patients. Our study also found that the methylation level of SDC2 was significantly higher in the faeces of patients with CRC than in normal patients, while the methylation level of adenocarcinoma was > AA > AD > polyp/normal.

The omission rates of CRC in negative patients in the high-risk and physical examination groups in this study were 5.10% and 3.40%, respectively, and it is presumed that SDC2 gene expression was lower, or methylation was not expressed in certain sources of intestinal cancer [24], which needs to be demonstrated in further studies. A total of 168 negative patients were diagnosed with polyps, accounting for 13.10% (168/1285). Previous studies have shown that only 1% of serrated ADs in polyps have the potential to develop into CRC [25]. In this study, three cases of serrated ADs in elderly patients aged ≥50 years were detected, and two cases of serrated ADs in young patients aged <50 years were not detected, with a detection rate of 60% and PPV of 0.38%, respectively. This indicated that the mSDC2 test had a high detection rate for serrated ADs in elderly patients aged ≥50 years, and those in young patients aged <50 years were missed. This requires further validation owing to the small number of cases. In this study, many patients with negative results in the first mSDC2 test did not wish to undergo further colonoscopy and could not be included in the analysis. Most of the 1285 patients who underwent subsequent screening and follow-up had some clinical presentation, so the false negative rate for CRC among the 9219 negative patients who did not undergo subsequent examination should be much lower, and this needs to be confirmed in future research.

## 5. Conclusion

As a regional and retrospective study, this study has some shortcomings: (1) The mSDC2 test for CRC screening still has a certain false positive and false negative rate; (2) the number of early CRC patients and AA patients is small, and there is a certain loss rate in the process of follow-up observation; (3) there are problems such as selection bias, missing information in the process of information collection, and some positive patients did not undergo a further colonoscopy and (4) only the data of the population who underwent the mSDC2 test for the first time in Qingyuan People’s Hospital were collected in this study, so the representativeness of the efficacy of the mSDC2 test for the diagnosis of colorectal tumours is insufficient. We are looking for the more frequent use of the faecal exfoliated cells mSDC2 test in the future for CRC screening and to provide a new non-invasive early screening technology for colorectal tumour prevention. At the same time, other multicentre, large-sample, prospective cohort studies are needed to provide a more detailed and comprehensive assessment of the performance of the mSDC2 test for colorectal tumours across regions, sexes, ages and populations with different risks.

## Data Availability

The datasets used and/or analysed during the current study are available from the corresponding author upon reasonable request.
